# Enhancing Thermal Conductivity and Photo-Driven Thermal Energy Charging/Discharging Rate of Annealed CMK-3 Based Phase Change Material

**DOI:** 10.3390/nano9030364

**Published:** 2019-03-05

**Authors:** Yanfeng Chen, Cuiyin Liu, Yue Situ, Jian Liu, Hong Huang

**Affiliations:** 1 School of Chemistry and Chemical Engineering, South China University of Technology, Guangzhou 510640, China; chen_yf@163.com (Y.C.); liu_cy@126.com (C.L.); situyue@scut.edu.cn (Y.S.); 2 Guangdong Provincial Key Laboratory of Distributed Energy Systems, School of Chemical Engineering and Energy Technology, Dongguan University of Technology, Dongguan 523808, China

**Keywords:** solar thermal systems, phase change material, annealed CMK-3, thermal conductivity, photo-driven charging and discharging rate

## Abstract

In this work, the CMK-3 is successfully prepared with SBA-15 as the template and first annealed to 2000 °C to improve thermal conductivity. The annealed CMK-3 has a thermal conductivity of 6.981 W m^−1^ K^−1^ higher than un-annealed CMK-3. The annealed CMK-3 is used to encapsulate the RT44HC, and RT44HC/annealed CMK-3 has 10-fold of thermal conductivity and enhanced thermal stability than RT44HC. The RT44HC/annealed CMK-3 has a large melting enthalpy of 177.8 J g^−1^ and good thermal stability. The RT44HC/annealed CMK-3 has optical absorptive coefficient of visible range of solar spectrum, which identify seven-fold higher than RT44HC. The RT44HC/annealed CMK-3 has great photo-thermal performance, and the photo-driven energy charging and discharging rate of RT44HC/annealed CMK-3 is almost 30-fold larger than the RT44HC. The results show that the annealed CMK-3 is a great mesoporous carbon nanomaterial for phase change materials and the annealed CMK-3 based phase change material has great potential in solar thermal utilizations such as solar water heating system and solar heating building systems.

## 1. Introduction

Solar thermal utilization systems is the broadest mode of renewable energy such as solar heating water systems, solar heating building systems, and solar drying systems [1,2]. In solar thermal systems, the overall efficiency is decided by receiver efficiency of gathering the solar spectrum intensity and Carnot efficiency of transfer of the thermal energy to other media [3]. The receiver efficiency depends on the optical absorption property and heat capacity of absorbed layer of media, which could be defined as photo-thermal cells [4,5]. The photo-thermal cells with great extinction property and heat capacity have advantage of increasing the receiver efficiency. The Carnot efficiency depends on the obtained temperature of the photo-thermal cells since high temperature means high effective efficiency [6]. To gain high overall efficiency, the photo-thermal cells should have good optical absorption property to improve photo-driven performance and large heat capacity to store solar thermal energy. Further, the photo-thermal cells should have large thermal conductivity to enhance the heat transfer in solar thermal energy charging/discharging processes.

Phase change material (PCM), a kind of material with phase change enthalpy 14-fold larger than sensible heat capacity [7], has great potential to be used as the photo-thermal cells in solar thermal systems. In solar thermal utilization systems, the PCM should have good optical absorption property, large latent heat capacity, and thermal conductivity. However, the PCM has poor optical absorption property in the visible range of solar spectrum [8], and the PCM has low thermal conductivity [9], which restricts the application of PCM. To overcome the aforementioned defects, porous materials, especially carbon based materials are used to encapsulate the PCM into micro- or nano-pores to form phase change composite [10]. The carbon materials such as expanded graphite [11], nano-graphite [12], CNT [13], and 3D-carbon form [14,15,16], have enhanced optical absorption property and thermal conductivity, which have the advantage of improving the photo-thermal performance of PCM. The expanded graphite based PCM has large thermal conductivity and phase change enthalpy [17], while the micron-scale expanded graphite reflects a lot of solar spectrum, decreasing the photo-thermal performance. The CNT [13], CNT sponge [9], and array [18] based PCM have good optical absorption property and large thermal conductivity, but the CNT sponge and array could encapsulate the PCM ratio lower than 60%. It is pivotal to develop a carbon material based PCM with good optical absorption property, large latent heat capacity, and thermal conductivity for enhancing photo-thermal conversion performance.

CMK-3, a mesoporous carbon material with template of SBA-15, is a great robust material for encapsulating the PCM [19]. The huge surface tension of CMK-3 could restrict the PCM in the mesoporous strip along the limbs. Further, the CMK-3 could introduce solar light and reflect multiply in the narrow strip to increase the resonant absorptive property of PCM. In general, the CMK-3 based PCM has great potential in using as photo-thermal cells in solar driven process. However, the CMK-3 was synthesized by long train alcohol with SBA-15 as template, the thermal conductivity of CMK-3 is low which could not largely improve the thermal energy charging/discharging rate of PCM.

To improve the thermal conductivity of CMK-3, we using a simple way to annealing the CMK-3 at 2000 °C. The annealed CMK-3 has much larger thermal conductivity. Then the as-prepared annealed CMK-3 was used encapsulated in paraffin to form phase change composite. The annealed CMK-3 has good absorption property for paraffin, and the phase change composite has large phase change enthalpy, high thermal conductivity, and good reversible stability. The annealed CMK-3 based phase change composite also has great optical absorption property and photo-driven energy charging and discharging capacity, which is promising to be used in solar thermal systems.

## 2. Materials and Methods

### 2.1. Materials

Technical grade paraffin RT44HC (melting point T_m_ = 44 °C) was purchased from Hangzhou Ruhr Energy Science and Technology Co, Ltd., Hangzhou, China. Dodecane (AR) was purchased from Aladdin reagent. The SBA-15 was supplied by Nanjing XFNANO Materials Tech Co., Ltd., Nanjing, China. AlCl_3_ and furfuryl alcohol was purchased from Aladdin Co, Ltd., Seoul, Korea.

### 2.2. Preparation of A-CMK-3

**Figure 1:**
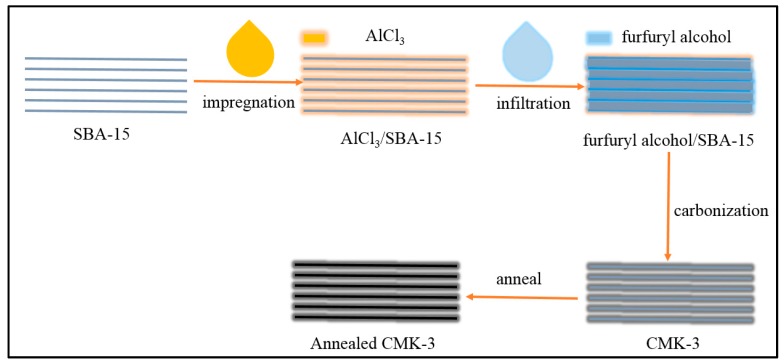
Preparation of annealed CMK-3.

The preparation of CMK-3 was followed and modified previous work [20], as shown in Figure 1. First, 20 g of SBA-15 was stirred for 12 h in a solution of AlCl_3_ (4 g) in ethanol, collected by filtration, and washed with ethanol for improving the carburization of SBA-15. Second, the furfuryl alcohol solution was introduced into the pores of SBA-15 with temperature increases from 80 to 300 °C at 1 °C min^−1^. Then, the temperature was increased to 850 °C with a rate of 5 °C min^−1^ and maintained at this temperature for 4 h to carbonize the furfuryl alcohol confined in SBA-15 to form CMK-3. Last, the temperature was increased to 2000 °C with a rate of 5 °C min^−1^ and maintained at this temperature for 4 h to anneal CMK-3 to form annealed CMK-3 (A-CMK-3). The samples were diluted with ethanol for three times and dried.

### 2.3. Preparation of A-CMK-3 Based PCM

The solid RT44HC was placed in a thermostat with temperature of 65 °C and melted to liquid, then, the A-CMK-3 was immersed into the liquid RT44HC to encapsulate the RT44HC. After certain minutes, the composites were refloated followed by filtered and dried, the RT44HC/A-CMK-3 was obtained. To evaluate the absorbability of A-CMK-3, different immersion time was conducted in the preparation process. To confirm the massive absorption portion of PCM in A-CMK-3, the composite with different mass fraction of PCM were placed on a filter paper, then were put into an oven with temperature of 70 °C. After heating for 2 h, the filter paper was observed whether the melted PCM leaking on the paper, and the weight of filter paper was measured to calculate the loss weight of PCM after heating.

### 2.4. Characterization and Measurement of A-CMK-3 Based PCM

The morphology and microstructure of A-CMK-3 and RT44HC/A-cmk-3 were observed on a field emission scanning electron microscopy (SU8020, Hitachi, Tokyo, japan). The hexagon pores of the A-CMK-3 was observed by a transmission electron microscopy (FEI Tecnai G20, Hillsboro, OR, USA). The structure of the composite was characterized by FT-IR spectra. The FT-IR spectra were recorded on a Bruker 550 from 400 to 4000 cm^−1^ using KBr pellets. The phase change temperature and latent heat of the samples were measured using a differential scanning calorimeter (Q20, TA). For DSC measurements, 5–8 mg for every sample was sealed in an aluminum pan for characterization at a heating rate of 10 °C min^−1^ under a constant stream of nitrogen at flow rate of 50 mL min^−1^. When conducting the DSC measurements, every samples were continuously tested by five times and the obtained results are calculated on average. The testing error of DSC measurement is ±2%. The thermal stability of A-CMK-3 and the RT44HC/A-CMK-3 was investigated by the thermogravimetric analysis (TGA) using a thermal analyzer (Q600 SDT, TA Instrument, New Castle, DE, USA). The measurements were conducted by heating the samples of 5–15 mg from room temperature to 600 °C at a heating rate of 10 °C min^−1^ under N_2_ atmosphere with a flow rate of 100 mL·min^−1^. The testing error of TGA measurement is ±1%. To test the stability of A-CMK-3 based PCM composite, 20 g of the composites were put on a temperature controlled chamber. The samples were conducted for 200 heating/cooling cycles with temperature of 20–70–20 °C. After 200 heating/cooling cycles, the melting/freezing temperature and enthalpy of composites was tested by DSC. About 10–12 g of RT44HC/A-CMK-3 powders were formed into a round block by dry pressing with a home-made cylindrical mold (4 cm inside diameter and 1 cm height) under the pressure of 100 kg cm^−2^. The packing density of RT44HC/A-CMK-3 was calculated of 771.3 kg m^−3^. The thermal conductivity of PCM block was measured using a thermal constants analyzer (Hot Disk TPS 2500S, Hot Disk AB, Göteborg, Sweden). The thermal conductivity measurement error is ±3%. For comparison purpose, RT44HC was melted and poured into the cylindrical mold to fabricate two pieces of casting samples so as to measure the thermal conductivity of RT44HC.

**Figure 2:**
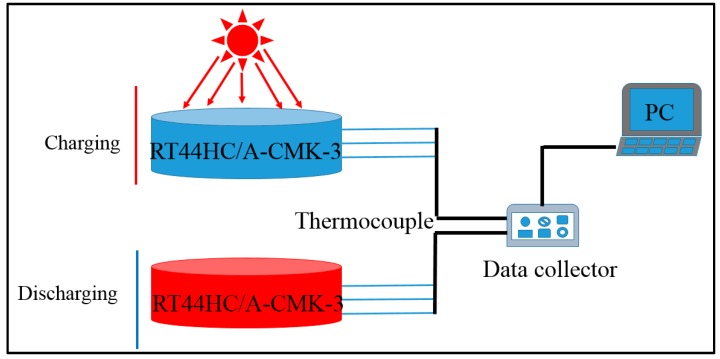
Photo-thermal charging and discharging performance of A-CMK-3 based phase change material (PCM).

The optical absorptive property of RT44HC/A-CMK-3 powders in wavelength range from 200 to 2200 nm was measured by PerkinElmer Lambda 950. The photo-thermal performance of RT44HC/A-CMK-3 block with weight of 10 g was conducted under simulated solar irradiation. As shown in Figure 2, the experimental apparatus consists of the photo-thermal conversion system and the data collection system. In this experiment, the samples were loaded in a quartzose beaker under the solar simulator (Microsolar300, Perfectlight), three thermocouple were inserted into the center of the PCM block with an interval height of 5 mm. The heat storage was carried out when the light was turned on. After the temperatures of the samples reached ~60 °C, the light was turned off and the samples were immediately cooled to room temperature, during which the samples under heat release process. The temperature of the samples during these periods were recorded by Agilent 30970A.

## 3. Results

### 3.1. Characterization of A-CMK-3

**Figure 3:**
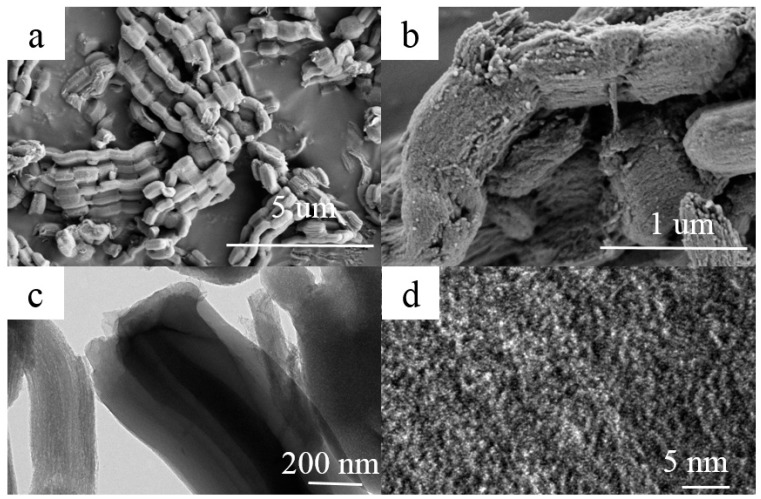
SEM (**a**,**b**) and TEM (**c**,**d**) images of A-CMK-3.

The morphology and microstructure of A-CMK-3 are observed by the SEM and TEM, as shown in Figure 3. The ordered mesoporous carbon CMK-3 was synthesized by replication using SBA-15 as the template and sucrose as the carbon source. A ‘‘German sausage’’ structure typical for A-CMK-3 alike to SBA-15 was observed in SEM micrographs taken at high magnification (Figure 3a,b) [21]. It can be seen that A-CMK-3 exhibits well-organized striped pores paralleling to each other. The TEM images of A-CMK-3 viewed perpendicular to the direction of the hexagonal pore arrangement is shown in Figure 3c,d. The white lines are corresponding to the mesoporous generated in the space previously occupied by the walls of SBA-15 template.

**Figure 4:**
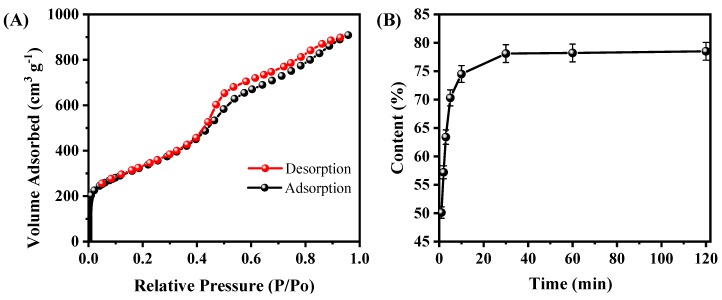
N_2_ adsorption/desorption isotherms of A-CMK-3 (**A**) and absorbability of A-CMK-3 with different impregnating time (**B**).

To further the microstructure of A-CMK-3, the N_2_ adsorption/desorption isotherm of A-CMK-3 was conducted. As shown in Figure 4A, the N_2_ adsorption/desorption curve is a type IV curve of mesoporous materials with a steep hysteresis loop. The sharp rise at relative pressure (P/P_0_) of about 0.4 indicates the existence of mesopores with narrow pore size distribution. According to the PCM, the BET surface area (S_BET_), pore volume (V_pore_), and pore size (D_pore_) are estimated to be 1164.61 m^2^ g^−1^, 1.45 cm^3^ g^−1^, and 2.05 nm, respectively. The surface area of A-CMK-3 is much larger than SBA-15 since the surface of A-CMK-3 is fulfilled with unordered micropores, which is produced by carbonization of the precursor [22]. The large surface area of A-CMK-3 has an advantage of improving the absorptive capacity of organic compounds. In order to measure the absorbability of A-CMK-3, adsorption time was tested. Figure 4B illustrates the mass fraction of RT44HC absorbed in the A-CMK-3 with different adsorption time according to the ratio of enthalpy of RT44HC/A-CMK-3 composite and pure RT44HC by DSC. The mass fraction of RT44HC increases with the adsorption time, specifically, the mass fraction rapidly increases from 0 to 74.5% as the adsorption time is increased from 0 to 10 min. When the adsorption time ranges from 10 to 30 min, the mass fraction of RT44HC gently increases from 74.5% to 78.6%. The RT44HC tends to a stationary mass ratio of 78.6% when keep increasing the adsorption time, implying that this stationary value is the maximum adsorption ratio of the A-CMK-3. To confirm the massive fraction of RT44HC, the leakage test of composites were observed on the filter paper. The composites with mass fraction of 77% and 78.6% did not show leakage in the paper, as the weight of filter paper did not increase. While the composite with mass fraction of 79% shows slight leakage in the paper, the weight of filter paper increased by 0.05 g, implying that the mass faction of composite is 78.6%. The absorption capacity of A-CMK-3 is higher than the un-annealed CMK-3 and other mesoporous SBA-15 and MCM-41, which has the absorption capacity lower than 70% [23,24]. The composite with high mass fraction of RT44HC has an advantage of enhancing the thermal energy storage performance.

### 3.2. Characterization of A-CMK-3 Based PCM

**Figure 5:**
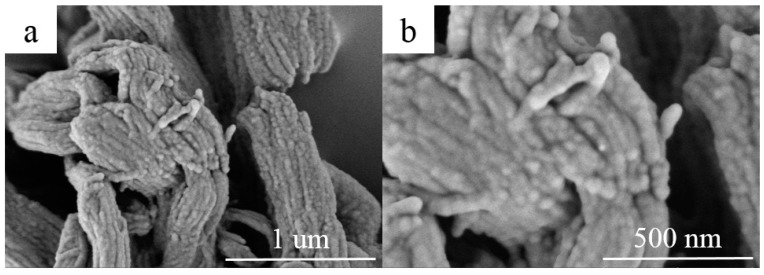
SEM images of A-CMK-3 based PCM (**a**,**b**).

The morphology of RT44HC/A-CMK-3 are observed by the SEM, as shown in Figure 5. The ordered striped pores of A-CMK-3 are filled with RT44HC, the tube structure was retained after impregnation with RT44HC. The composite maintains as ‘‘German sausage’’ structure and is inflated. The A-CMK-3 owns the large capillary force and surface tension to immerse the paraffin into the inner and surface space which reveals good thermal and chemical stability of A-CMK-3.

**Figure 6:**
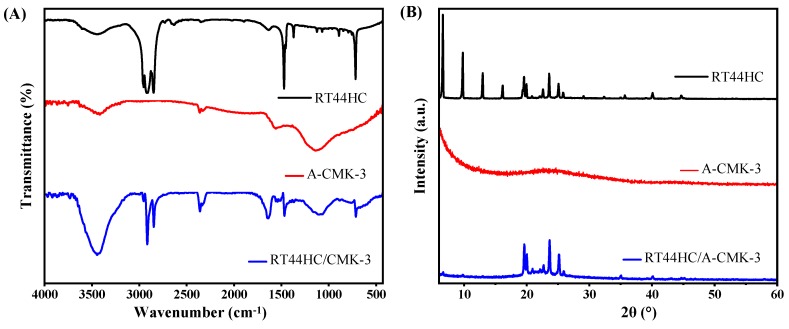
FT-IR spectrum (**A**) and XRD pattern (**B**) of A-CMK-3 based PCM.

To further confirm the tube structure of RT44HC/A-CMK-3, the FT-IR spectrums are conducted. The FT-IR spectra of RT44 HC, A-CMK-3 and RT44HC/A-CMK-3 are displayed in Figure 6A. In the spectrum of RT44HC, the peaks at 2956 and 2917 cm^−1^ correspond to the stretching vibration of –CH_3_, and 2850 cm^−1^ is ascribed to the stretching vibration of –CH_2_–. The peaks at around 1465 cm^−1^ belong to the deformation vibration of –CH_2_ and –CH_3_, and the peak at 720 cm^−1^ is due to the in-plane rocking vibration of –CH_2_ [25]. The spectrum of the A-CMK-3 shows bands that are assigned to the vibrations of the carbon structure, namely the band at 1565 cm^−1^ assigned to C=C stretching vibrations of the aromatic carbon and the band centered at 1130 cm^−1^ which has contributions from the skeletal C=C tangential motions. The band of low intensity at 3400 cm^−1^ is assigned to O–H stretching vibrations from phenol groups [26]. In the spectrum of RT44HC/A-CMK-3, the peaks assigned to A-CMK-3 at 3400, 1565, and 1130 cm^−1^, and the peaks are assigned to paraffin at 2956, 2917, 2850, 1465, and 720 cm^−1^ still existed, and no significant new peak is observed, which confirms that the tube structure of the RT44HC/A-CMK-3, also reveals the composite PCM are physical interaction.

The XRD patterns of the RT44HC, A-CMK-3, and RT44HC/A-CMK-3 composite are displayed in Figure 6B. In the pattern of RT44HC, the strong diffraction peaks at 2*θ* = 6.67, 9.78, 12.974, 16.17, 19.57, 19.95, 23.57, and 25.08° were caused by regular crystallization of the pure RT44 HC, which are attributed to the diffractions of (110), (200), and other crystal planes [27]. In the pattern of A-CMK-3, there is no sharp diffraction peaks from 5 to 60°. The XRD pattern of the RT44HC/A-CMK-3 composite contains all the peaks of RT44HC and no new peaks appear, whereas intensity of the peaks are relatively lower in comparison with RT44HC, especially the intensity of peaks at 2*θ* = 6.67, 9.78, 12.974, and 16.17° are much lower than the peaks of RT44HC, implying that the RT44HC is confined in the narrow space, the small crystals could not generated in the narrow space [28]. The XRD results verifying that the RT44HC is confined in the space of A-CMK-3.

### 3.3. Thermal Conductivity of A-CMK-3 Based PCM

**Figure 7:**
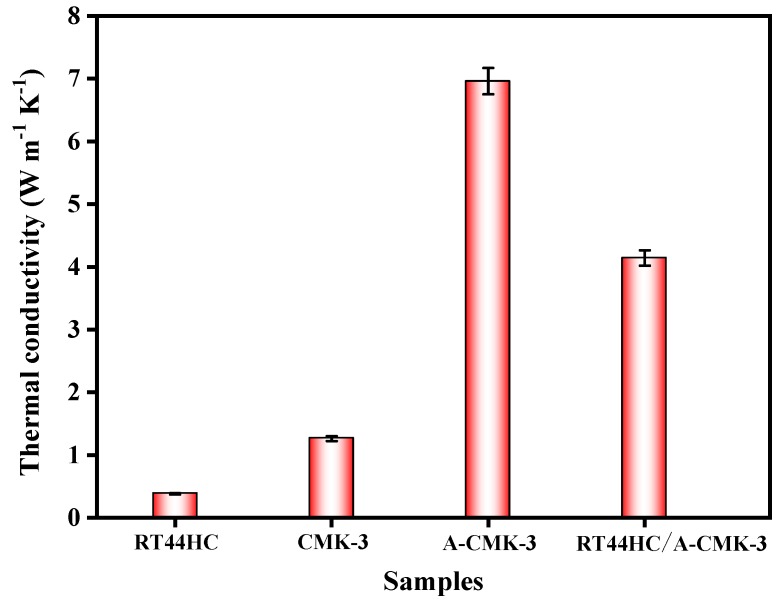
Thermal conductivity of A-CMK-3 based PCM.

The thermal conductivity of the RT44HC and RT44HC/A-CMK-3 block is measured. As shown in Figure 7, the thermal conductivity of the RT44HC casting sample with the density of 900 kg m^−3^ is 0.380 W m^−1^ K^−1^, the thermal conductivity of CMK-3 block is 1.262 W m^−1^ K^−1^. After annealing work, the thermal conductivity of A-CMK-3 block is 6.981 W m^−1^ K^−1^, which is 4.5-fold larger than the un-annealed one. The thermal conductivity of the RT44HC/A-CMK-3 block is 4.241 W m^−1^ K^−1^ as the packing density of 771.3 kg m^−3^. The thermal conductivity of RT44HC/A-CMK-3 block is lower than the A-CMK-3 since the RT44HC adhere to the surface of A-CMK-3, decreasing the thermal conductivity. Note that the RT44HC/A-CMK-3 block have 10-fold higher thermal conductivity than the RT44HC, owing to the integration of RT44HC with A-CMK-3 that has superior thermal conductivity. It is indicated that the annealed A-CMK-3 based PCM has an advantage of gaining large thermal conductivity, since annealed A-CMK-3 has much better purity than the un-annealed one. High temperature annealing work could largely increase the thermal conductivity of the 3D carbon framework, which is identical with previous work [29], which has thermal conductivity of 3.7 W m^−1^ K^−1^ in annealed carbon block based PCM composites. The A-CMK-3 has much higher thermal conductivity than the un-annealed CMK-3 since the residual N and O element embedded in the carbon layer of CMK-3. In this work, we using a simple way to fabricate a porous A-CMK-3 with higher thermal conductivity by remove the residual N and O element, with based PCM has great potential in thermal energy charging/discharging behavior.

### 3.4. Melting and Freezing Behavior of A-CMK-3 Based PCM

**Figure 8:**
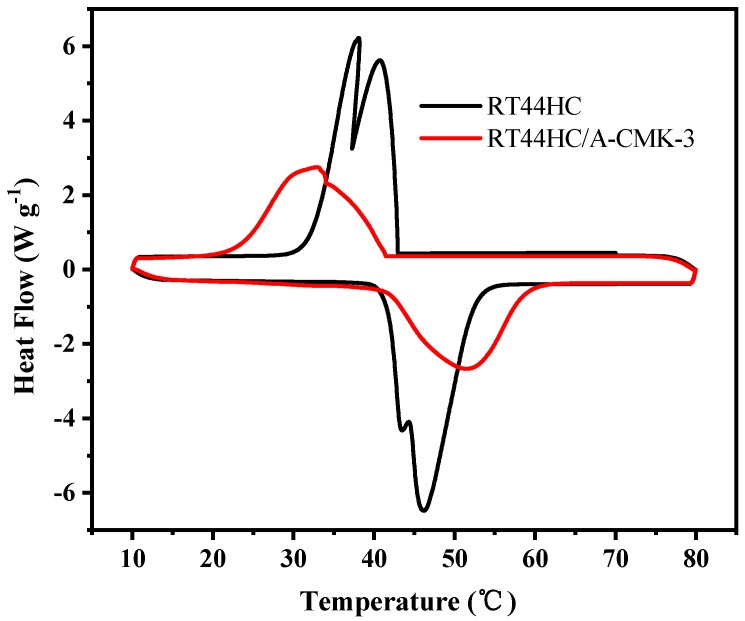
Melting and freezing behavior of A-CMK-3 based PCM.

The phase change temperature and latent heat of RT44HC and the RT44HC/A-CMK-3 were measured. Figure 8 shows the melting and freezing curves of the RT44HC and the RT44HC/A-CMK-3. For the pure RT44HC, there are two endothermic peaks in the melting DSC curves and two exothermic peaks in the solidifying DSC curves. It is ascribed to different crystal transitions according to the XRD patterns. The melting and freezing temperature is 40.97 and 41.52 °C for the pure RT44HC and 40.99 and 41.63 °C for RT44HC/A-CMK-3 and the melting and freezing latent heat is 226.1 and 223.5 J g^−1^ for the pure RT44HC and 177.8 and 173.1 J g^−1^ for RT44HC/A-CMK-3, respectively. The phase change characteristics of the RT44HC/A-CMK-3 are quite similar to those of pure RT44HC, because there is no chemical reaction between RT44HC and A-CMK-3 in preparation process. The encapsulation ratio (*R*) of RT44HC by A-CMK-3 can be calculated by the results of the DSC measurements and according to Equation (1).

(1) $$R=\frac{\Delta H_{m,Composite}}{\Delta H_{m,Paraffin}}\times 100\%,$$

where Δ*H_m,Composite_* and Δ*H_m,Paraffin_* represent the melting latent heat of RT44HC/A-CMK-3 and RT44HC, respectively. The encapsulation ratio (*R*) of paraffin in the composite PCMs is calculated to be 78.6%.

### 3.5. Thermal Stability of A-CMK-3 Based PCM

**Figure 9:**
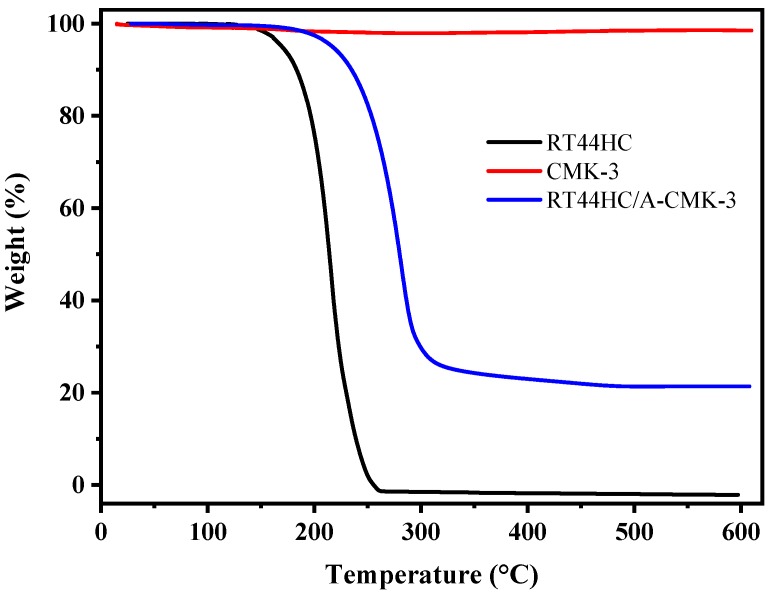
Thermal stability of A-CMK-3 based PCM.

The thermal stability of RT44HC, A-CMK-3, and RT44HC/A-CMK-3 are measured by TGA and the curves of weight loss percentage are shown in Figure 9. The RT44HC starts to be removed at about 140 °C, and the final weight loss percentage is nearly 100% at 260 °C. For A-CMK-3, there is no obvious weight loss till 600 °C. In the TGA curve of the RT44HC/A-CMK-3, the composite starts to lose weight at ~160 °C and the final weight loss percentage is nearly 78.6%. The TGA measurement also reveals that the weight percentage of the RT44HC is ~80%, matching with the result of DSC measurement, and the TGA results also indicate that the composite PCMs could slightly enhance the thermal stability of the RT44HC.

### 3.6. Heating/Cooling Stability of A-CMK-3 Based PCM

**Figure 10:**
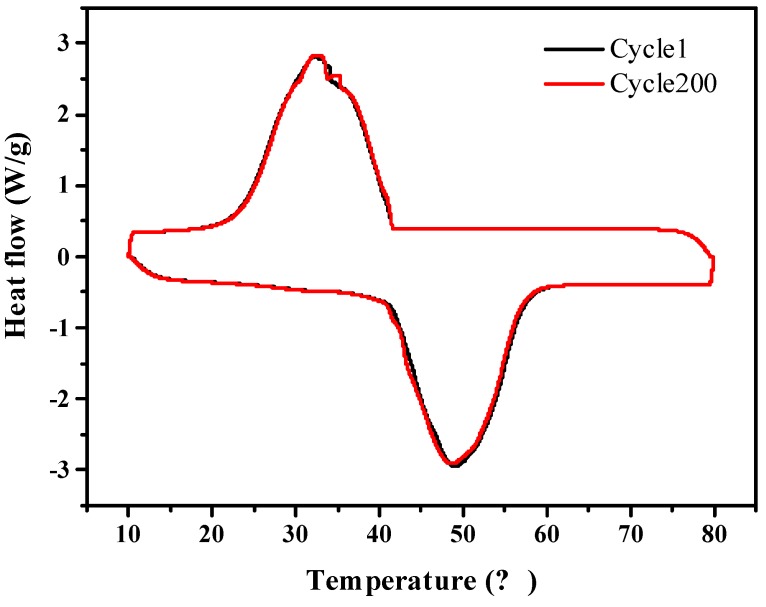
Heating/cooling stability of A-CMK-3 based PCM composite.

Figure 10 shows that the A-CMK-3 based PCM has melting/freezing temperature of 40.97 and 41.65 °C, and melting/freezing enthalpy of 176.6 and 172.1 J g^−1^. As compared to melting/freezing temperatures of 40.99 and 41.63 °C and the melting/freezing enthalpy are 177.8 and 173.1 J g^−1^ for RT44HC/A-CMK-3 before heating/cooling cycles. The relative deviation of composite before and after 200 heating/cooling cycles is lower than 0.6%, indicating that the composite has great stability.

### 3.7. Optical Absorptive Property of A-CMK-3 Based PCM

**Figure 11:**
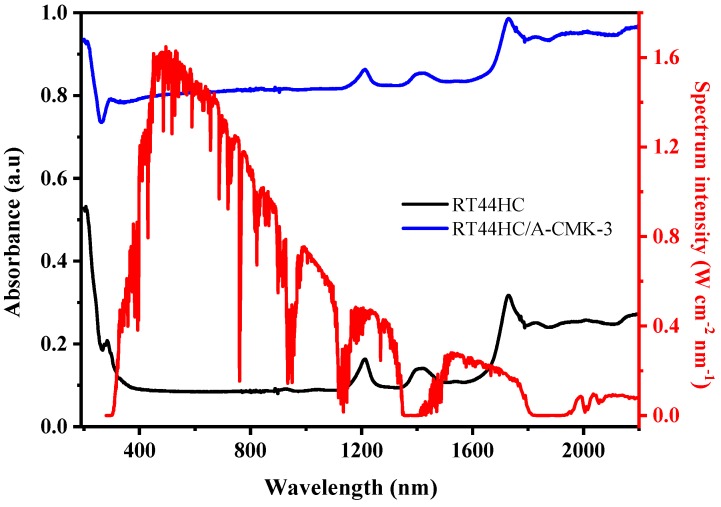
Optical absorptive property of A-CMK-3 based PCM.

The optical extinction property of PCM is shown in Figure 11. The absorbance of RT44HC is 0.1 in the visible range and increases to 0.3–0.4 in the infrared range. It can be clearly seen that RT44HC has poor absorption property in whole range of solar spectrum. The absorbance of RT44HC/A-CMK-3 is increased to 0.8 in the visible range and increased to 0.82–0.98 in the infrared range, which is seven-fold larger than RT44HC in the visible range and two-fold larger than paraffin in the infrared range. It is clearly seen that the solar radiation intensity of visible range occupies 52% of the whole range of solar spectrum intensity, the absorbance property of RT44HC/A-CMK-3 in the visible range is much higher than RT44HC, which is advantage of gathering solar spectrum intensity of PCM.

### 3.8. Photo-Thermal Charging and Discharging Property of A-CMK-3 Based PCM

**Figure 12:**
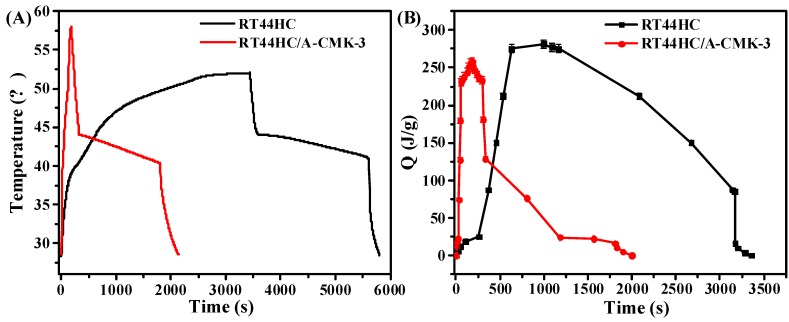
Photo-thermal performance of PCM (**A**), thermal energy charging and discharging behavior of PCM (**B**).

The photo-thermal performance of PCM is shown in Figure 12. In the heating process, it takes 260 s for RT44HC to increase the temperature from 28 to 40 °C, then takes 1668 s to melt the RT44HC as the temperature increase from 40 to 50 °C. For RT44HC/A-CMK-3, it takes 24s to increase the temperature from 28 to 40°C, and takes 40s to melt the PCM, as shown in Figure 12A. It took thirty times for RT44HC/A-CMK-3 compared to RT44HC in the heating process, indicating that adding A-CMK-3 could largely improve the photo-thermal performance of PCM, The A-CMK-3 is a good photon trapping material as the resonance of π-electronic. In the cooling process, it takes 2400 s for paraffin to decrease the temperature from 75 to 30 °C, and takes 3200 s for RT44HC to decrease the temperature from 50 to 28 °C. The cooling time of RT44HC/A-CMK-3 is 1900 s, which is 500 s less than the RT44HC since the thermal conductivity of RT44HC/A-CMK-3 is larger than the RT44HC. It is indicated that the RT44HC/A-CMK-3 could largely improve the photo-thermal performance. To further evaluate the photo-thermal performance of PCM, The thermal energy charging capacity of PCM is calculated as:

(2) $$Q_{T}=\int_{T=28}^{T=50}CpdT+\Delta H_{m},$$

where the *Q_T_* is thermal storage capacity of PCM, m is the mass of PCM, *C_p_* is the heat capacity of PCM. Δ*H_m_* is the enthalpy of PCM. As shown in Figure 12B, the thermal energy charging capacity of RT44HC increased to 270 J in 750 s, and increased to 250 J in 64 s for RT44HC/A-CMK-3, which is one-eleventh of RT44HC, indicating that the A-CMK-3 could largely improve the photo-thermal energy charging and discharging rate of PCM. The A-CMK-3 based PCM has great photo-thermal performance and effective thermal storage capacity.

### 3.9. Reversible Property of A-CMK-3 Based PCM

**Figure 13:**
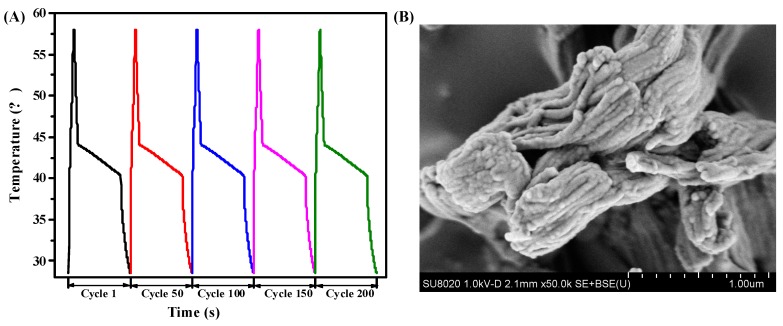
Photo-thermal performance of PCM at different cycles(A) and SEM image of PCM after 200 cycle.

To evaluate the reversible stability of RT44HC/A-CMK-3, the photo-thermal performance with different cycles was conducted, as shown in Figure 13A. The result shows that the RT44HC/A-CMK-3 has similar increasing and decreasing temperature trends with different cycles, implying the RT44HC/A-CMK-3 has excellent reversible stability. To further verify the stability of RT44HC/A-CMK-3, the morphology of RT44HC/A-CMK-3 before and after heating and cooling cycles are measured. As shown in Figure 13B, the morphology and microstructure of RT44HC/A-CMK-3 after 200 cycles is similar with that before heating and cooling cycles, verifying the RT44HC/A-CMK-3 has great reversible stability.

## 4. Conclusions

In this work, a mesoporous carbon material annealed CMK-3 was successfully prepared and used to encapsulate the RT44HC to form nano-PCM. The annealed CMK-3 has a thermal conductivity of 6.981 W m^−1^ K^−1^, which is 4.5-fold higher than the un-annealed CMK-3. The annealed CMK-3 was used to encapsulate the paraffin RT44HC, the RT44HC/A-CMK-3 has 10-fold the thermal conductivity and enhanced thermal stability than RT44HC. The RT44HC/A-CMK-3 has seven-fold higher optical absorption properties than RT44HC in the visible range. The RT44HC/A-CMK-3 has great photo-thermal performance, and the photo-driven energy charging and discharging rate of RT44HC/A-CMK-3 is almost 30-fold larger than the RT44HC. The results show that the A-CMK-3 is a great mesoporous carbon nanomaterial for PCM and the A-CMK-3 based PCM has great potential in solar thermal utilizations such as solar water heating system, solar heating building systems.
